# Study of the *MTHFR* 677C>T Polymorphism in Children and Adolescents with Hashimoto’s Thyroiditis: An Original Case–Control Study

**DOI:** 10.3390/diagnostics15111310

**Published:** 2025-05-23

**Authors:** Savvas Kolanis, Elisavet Georgiou, Eleni P. Kotanidou, Vasiliki Rengina Tsinopoulou, Evdoxia Sapountzi, Emmanouel Hatzipantelis, Liana Fidani, Assimina Galli-Tsinopoulou

**Affiliations:** 1 2nd Department of Paediatrics, School of Medicine, Faculty of Health Sciences, Aristotle University of Thessaloniki, AHEPA University Hospital, 54636 Thessaloniki, Greece; savvasks@auth.gr (S.K.); epkotanidou@auth.gr (E.P.K.); vitsinop@auth.gr (V.R.T.); sevdoxia@auth.gr (E.S.); hatzip@auth.gr (E.H.); 2 Laboratory of Biological Chemistry, School of Medicine, Faculty of Health Sciences, Aristotle University of Thessaloniki, 54124 Thessaloniki, Greece; elgeorgiou@auth.gr; 3 Laboratory of Medical Biology-Genetics, School of Medicine, Faculty of Health Sciences, Aristotle University of Thessaloniki, 54124 Thessaloniki, Greece; sfidani@auth.gr

**Keywords:** Hashimoto thyroiditis, methylenetetrahydrofolate reductase, *MTHFR* gene, single nucleotide polymorphism, anti-TPO, anti-TG, child, adolescent

## Abstract

**Background/Objectives**: Hashimoto’s thyroiditis (HT) is the most common cause of hypothyroidism during childhood and adolescence. Children and adolescents with HT have an increased susceptibility to the development of thyroid nodules and thyroid cancer. Among the genetic causes of thyroid cancer, the 677C>T polymorphism of the methylenetetrahydrofolate reductase (*MTHFR*) gene is also reported. This study investigated for the first time the association between the 677C>T polymorphism (rs1801133) of the *MTHFR* gene and HT in children and adolescents. **Methods**: This case–control study included 130 children and adolescents with HT and 130 healthy controls. The 677C>T polymorphism of the *MTHFR* gene was studied in all participants with Restriction Fragment Length Polymorphism (RFLP) methodology for genetic variance analysis. **Results**: Children and adolescents with HT presented approximately 2.5 times more frequently the T allele sequences (CT and TT variants) and the T alleles in total for the 677C>T polymorphism of the *MTHFR* gene compared to the healthy population (OR: 2.56, CI: 1.53–4.21 and OR: 2.57, CI: 1.59–4.16, respectively). Children and adolescents with HT and T allele sequences (CT and TT variants) exhibited abnormal thyroglobulin antibodies (anti-TG) two times more frequently compared to those with the wild-type (CC) sequence in the same population (OR: 2.13, CI: 1.04–4.389). **Conclusions**: Children and adolescents with HT showed an increased frequency of T allele sequences (CT and TT variants) and total T alleles of the 677C>T polymorphism of the *MTHFR* gene compared to the healthy population.

## 1. Introduction

Hashimoto’s thyroiditis (HT) is an autoimmune disease of the thyroid gland, which ultimately leads to the destruction of the gland. It is the most common form of thyroiditis in countries with dietary iodine sufficiency. The incidence of the disease in childhood is reported to be 1.2%, with a higher frequency in adolescence [1,2,3]. The pathophysiology of the disease involves an autoimmune condition in which thyroid peroxide (anti-TPO) and thyroglobulin (anti-TG) autoantibodies cause lymphocytic infiltration of the thyroid gland, which often presents as goiter. The etiology of the disease involves the coexistence of both genetic and environmental factors [2,4,5,6,7,8].

Sporadic thyroid nodules in children are rare (prevalence 0.2–5.1%), with a significant increase observed among adolescents and young adults (up to 7–13%) [9,10]. Despite the reduced prevalence of thyroid nodules in children and adolescents, there is an increased incidence of their malignant transformation (22–26%) compared to adults (5%) [10,11,12]. Moreover, thyroid gland nodules coexist with HT in a slightly higher frequency among children and adolescents (31.5%) compared to the adult population (27%) [9,13,14]. Thus, investigation of thyroid nodules in children and adolescents is of utmost importance in order to exclude possible malignant transformation [9,14,15].

Frequency of thyroid gland malignancy among children with HT is reported to range from 0.67% to 3%, a percentage which is substantially higher than its frequency in the general population (thyroid gland cancer frequency in children: 0.02%, incidence: 1.14/100,000 per year) [12,16]. Additionally, 23.2% of patients who developed papillary thyroid carcinoma also presented with coexisting HT % [17,18], a significantly higher prevalence when compared to the general population, where HT is estimated to affect approximately 5% [19].

Thyroid cancer is the most frequent cancer of the endocrine system (incidence 90%) and it accounts for 2% of all human cancers. It is the sixth most common cancer in females and is three times more frequent compared to males. In addition, it is also the most common endocrine cancer of childhood with an incidence of approximately 6%. Its overall 5-year survival rate is 98% [16,20]. The etiology of thyroid cancer in children is multifactorial. An increased risk is recorded in childhood cancer survivors after radiotherapy or chemotherapy, as well as in tumor growth syndromes such as hamartomas and familial adenomatous polyposis syndrome [16]. Mutations and polymorphisms in the *BRAF*, *RET*, *NTRK*, *ALK*, *DICER1*, *TERT*, *TP53*, *PTEN*, *PIK3CA* and *MTHFR* genes are associated with papillary thyroid carcinoma, while mutations in the *RAS* and *PAX-8-PPARg* genes are associated with follicular thyroid cancer [21,22,23,24,25].

The metabolism of folic acid involves methylation, synthesis and various repair processes of deoxyribonucleic acid (DNA). Abnormalities found in folate metabolism affect gene expression and DNA stability. Methylenetetrahydrofolate reductase (*MTHFR*) is an enzyme of utmost importance in folate metabolism, as it affects DNA methylation and is responsible for the irreversible reduction of 5,10 methyltetrahydrofolate to 5 methyltetrahydrofolate. In addition, it is used as a co-substrate for the remethylation of homocysteine to methionine [26,27,28].

The 677C>T (rs1801133) is the most frequent polymorphism of the *MTHFR* gene and is related to reduced reductase activity (down to 50%) [29]. Specifically, the change of cytosine to thymine at nucleotide position 677 of the gene results in the replacement of alanine with the amino acid valine. This causes reduced activity of reductase enzyme and, consequently, reduced levels of folic acid in the plasma, as well as increased levels of homocysteine [29,30,31]. The 677C>T polymorphism is associated with increased cancer risk and other diseases and conditions such as stroke, heart disease, diabetes mellitus, hypothyroidism, autism spectrum disorders and congenital disorders [25,32,33,34,35,36,37,38,39,40,41].

The risk of developing thyroid cancer is reported to be increased among patients diagnosed with HT [17]. *MTHFR* gene mutations are associated with the development of thyroid cancer [25]. The 677C>T polymorphism of the *MTHFR* gene is a known cause of abnormally increased levels of homocysteine [27]. Hyperhomocysteinemia is generally associated with the development of various types of cancer in humans and, additionally, elevated homocysteine serum levels are reported in patients with HT [42,43]. Additionally, 677C>T polymorphism is associated with increased risk of hypothyroidism [38]. However, no investigation has been reported so far about the association of the 677C>T polymorphism of the *MTHFR* gene and HT in children and adolescents.

This study investigated for the first time the association between the 677C>T (rs1801133) polymorphism of the *MTHFR* gene and HT in children and adolescents.

## 2. Materials and Methods

The present case–control study was designed in accordance with the Strengthening the Reporting of Observational Studies in Epidemiology (STROBE) guidelines [44]. The participants’ selection took place at the Second Department of Pediatrics, AHEPA University General Hospital in Thessaloniki, Greece, from September 2020 until March 2024. The process included random selection of children and adolescents that met the eligibility criteria and visited the Department either for routine check or hospitalization.

This study’s inclusion criteria for the patient group consisted of children and adolescents with an age range of 4–18 years diagnosed with HT. The diagnosis of HT was verified by abnormal anti-TPO titer values and/or abnormal anti-TG titer values at the time of the selection process or in the past. Participants were excluded from the patient group if they presented history of confirmed diagnosis of cancer or chronic or systemic disease or autoimmune disease, either at the time of the selection process or in the past. The control group included healthy children and adolescents with a similar age range of 4–18 years. Participants were excluded from the control group if they presented history of confirmed diagnosis of cancer, chronic or systemic disease or autoimmune disease or family history of HT, either at the time of the selection process or in the past. The patient group included 130 children and adolescents with a mean age of 9.98 years, while the control group included 130 children and adolescents with a mean age 10.50 years. In the event of patient withdrawal from the study, participant selection continued until the sample was completed. Thorough anamnesis and physical examination were performed on all participants by the same experienced clinician. Peripheral blood was obtained from all participants for both genetic and biochemical analysis. All participant samples concerning the laboratory variables were assessed at AHEPA University General Hospital, Thessaloniki, Greece.

The measurement of thyroid gland hormones (TSH and fT4) and thyroid gland autoantibodies (anti-TPO and anti-TG) were performed by the electrochemiluminescent immunoassays (ECLIA) method. All measurements were performed on the Roche COBAS 8000 analyzer system (biochemical and immunological) (Roche Diagnostics GmbH, 68305 Mannheim, Germany).

Genomic DNA (gDNA) was isolated from 100 μL of peripheral blood, using the Monarch Genomic DNA Purification Kit (Cat #T3010, New England Biolabs, Ipswich, MA, USA), according to the manufacturer’s protocol. The quality and quantity of the isolated gDNA was assessed by spectrophotometry and electrophoresis.

**Figure 1:**
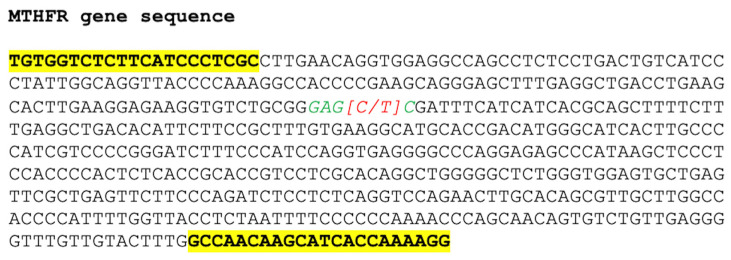
The 513 bp fragment of the *MTHFR* gene amplified by PCR. The sequences for primers annealing are bold and highlighted. The sequence recognized by the restriction endonuclease is shown in italics and green letters, while the single nucleotide variant position is shown in brackets and in red.

For the genetic variant analysis, Restriction Fragment Length Polymorphism (RFLP) was applied [45]: a 513 bp (base pair) fragment of the *MTHFR* gene, containing the 677C>T variant site, was amplified by polymerase chain reaction (PCR). PCR was carried out using KAPA HiFi polymerase (Kapa Biosystems, Cape Town, South Africa) and the following primers: forward 5′-TGTGGTCTCTTCATCCCTCGC-3′ and reverse 5′-CCTTTTGGTGATGCTTGTTGGC-3′ (Figure 1).

**Figure 2:**
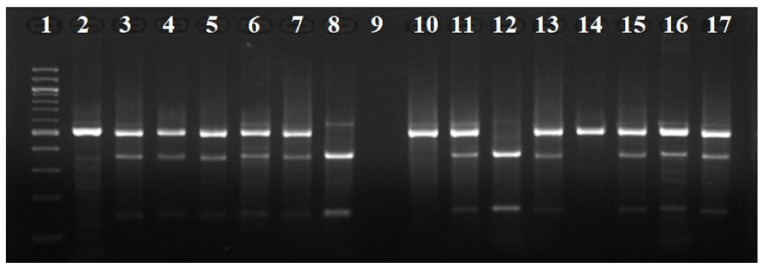
Agarose gel electrophoresis of the digestion products of the 677C>T polymorphism of the *MTHFR* gene from our study. Lane 1: molecular weight index (100 bp ladder). Lanes 2, 10, 14: samples homozygous for the C allele (CC). Lanes 8, 12: samples homozygous for the T allele (TT). Lanes 3, 4, 5, 6, 7, 11, 13, 15, 16, 17: samples heterozygous (CT). Lane 9: No samples were used.

Cycling conditions included an initial denaturation step at 95 °C for 3 min, followed by 35 cycles of denaturation at 98 °C for 20 s, annealing at 63 °C for 15 s and extension at 72 °C for 15 s. The PCR products were then digested with restriction endonuclease Hinf I (Cat #R0155, New England Biolabs, Ipswich, MA, USA). The reaction mixture was incubated at 37 °C for 30 min, followed by incubation at 80 °C for 20 min, to inactivate the enzyme. The digestion products were electrophoresed on a 1.5% agarose gel and DNA fragments were visualized on a UV light bank, using ethidium bromide staining (Figure 2).

Sample size calculation was performed with G power Calculator (GPower 3.1) and Genetic Association Study (GAS) Power Calculator (accessed on 1 August 2020). The parameters included the frequency of the under-investigation polymorphism, the prevalence of the investigated disease, the desired study power (80%) and the relative risk (RR). Statistical methods were used depending on the disease model (multiplicative, recessive, dominant, additive, homozygous, allelic). The odds ratio (OR) was calculated along with the 95% confidence interval. In cases of zero frequencies, a correction was applied by adding 0.5, and for multiple comparisons, the false discovery rate (FDR) correction was used.

The IBM SPSS 30.0 statistical software (IBM Inc., Armonk, NY, USA) was used for statistical analysis. The description of qualitative variables was presented with percentages and frequencies. Quantitative variables were described using means and standard deviations or using medians and interquartile range, depending on normality of the distribution of the explored variable (Kolmogorov–Smirnov test). The relationship between qualitative variables was examined for statistical independence using Pearson’s chi-square test or Fisher’s exact test. For the relationship of quantitative variables between two independent groups, the independent samples *t*-test or the Mann–Whitney U test was used, while the relationship of quantitative variables between three or more independent groups was performed by using the one-way ANOVA test or Kruskal–Wallis H test. In case of a statistically significant result, repeated tests (post hoc tests) were performed by applying Tukey’s correction or the Bonferroni correction. Linear regression models were used to correlate parameters between groups, also considering other factors such as age, gender, etc. All tests performed were two-sided at a 5% statistical significance level.

## 3. Results

Baseline characteristics of the participants in this study are presented in Table 1.

The primary objective was to examine the possible correlation of the 677C>T (rs1801133) polymorphism of the *MTHFR* gene and HT in children and adolescents. Children and adolescents with HT exhibited a more than 2.5-fold increase in the T allele sequences (CT and TT variants) of the 677C>T polymorphism of the *MTHFR* gene when compared with healthy children and adolescents (OR: 2.56, CI: 1.53–4.21). Moreover, the CT variants of the 677C>T polymorphism appeared more than two times more frequently in the patient group compared to the control group (OR: 2.17, CI: 1.31–3.59). There was no statistically significant difference in the frequency of homozygous T allele sequences (TT variants) of the 677C>T polymorphism between the study groups (OR: 1.75, CI: 0.73–4.16). The total C alleles did not show a statistically significant difference between the two groups (OR: 0.49, CI: 0.21–1.15). However, the total T alleles also presented a greater than 2.5-fold increase in the patient group compared to the control group (OR: 2.57, CI: 1.59–4.16) (Table 2).

Further analysis of the 677C>T polymorphism (with the additive model) through linear regression analysis and a logistical regression analysis showed also a consistent and significant association with HT in children and adolescents (OR: 2.05, CI: 1.35–3.03). Furthermore, in these analyses, other variables included in this study showed no significant association with HT, besides the autoantibodies (Tables S1 and S2 in the Supplementary Materials).

The secondary objectives were mainly focused on the patient group. There was no statistically significant difference in the 677C>T polymorphism concerning mean age of the patient subgroups (subgroups based on the variants of the 677C>T polymorphism) (*p* value: 0.343), nor within the age groups of children and adolescents with HT (OR: 1.435, CI: 0.703–2.932) (Tables S3 and S4). There was also no statistically significant difference between the 677C>T polymorphism and gender groups in children and adolescents with HT (OR: 1.54, CI: 0.40–5.82) (Table S5). Additionally, no significant association was recorded between patients’ thyroid status (hypothyroidism, euthyroidism, hyperthyroidism) during the initial diagnosis and the frequency of the 677C>T polymorphism of the *MTHFR* gene in children and adolescents with HT (*p* value: 0.778).

There was no correlation between the 677C>T polymorphism and the titer (as mean value) of anti-TPO nor the anti-TG in children and adolescents with HT (*p* value: 0.357 and *p* value: 0.643, respectively) (Table 3).

There was also no statistically significant difference of the anti-TPO as dichotomous values (positive titers vs. negative titers) in children and adolescents with HT and the T allele sequences (CT and TT variants) compared with children and adolescents with HT and the wild type sequence (CC) (OR: 0.377, CI: 0.117–1.209). However, children and adolescents with HT and T allele sequences (CT and TT variants) presented approximately 2 times more frequent abnormal anti-TG antibodies as dichotomous values (positive titers vs. negative titers) when compared with children and adolescents with HT and the wild type sequence (CC) (OR: 2.13, CI: 1.04–4.389) (Table 4).

## 4. Discussion

The present case–control study revealed that children and adolescents with HT exhibited more frequent T allele sequences (CT and TT variants) of the 677C>T polymorphism of the *MTHFR* gene compared to the healthy population. Additionally, the CT variant of the 677C>T polymorphism also appeared more often in children and adolescents with HT when compared to the healthy population. Total T alleles are also present more often among children and adolescents with HT compared to the healthy population, while total C alleles did not differ between the studied groups. No significant association was recorded between the 677C>T polymorphism and age or gender of participants with HT. There was also no significant association between the 677C>T polymorphism and the titer of anti-TPO and the anti-TG autoantibodies or the thyroid status of patients at the time of initial diagnosis in children and adolescents with HT. However, anti-TG autoantibodies were found to be abnormal more frequently in children and adolescents with HT and T allele sequences (CT and TT variants) compared with children and adolescents with HT and the wild-type CC polymorphism of the 677C>T.

Many genetic factors have been associated with thyroid cancer across all ages [16,23], and among them, the 677C>T polymorphism of the *MTHFR* gene has been proposed as a possible underlying genetic defect in the context of thyroid malignancies. The meta-analysis by Yan et al. [25] examined the association between the 677C>T polymorphism and thyroid cancer, examining a total of 360 cases of thyroid cancer, concluding that the homozygous T (TT) variant and the total of T alleles are associated with increased susceptibility to thyroid cancer. This polymorphism, besides thyroid cancer, is also associated with other thyroid dysfunctions, such as hypothyroidism [38]. Additionally, another meta-analysis [38] involving a total of 817 patients, showed that the 677C>T polymorphism occurred at a significantly higher frequency in patients with HT compared to the healthy population.

The 677C>T polymorphism is known to lead to abnormally elevated serum homocysteine levels [27]. Hyperhomocysteinemia is an independent risk factor to the development of various types of cancer in humans [42]. Moreover, hyperhomocysteinemia is associated with an increased frequency of autoimmune diseases, including HT. In the same context, patients with HT and subclinical hypothyroidism exhibited significantly higher homocysteine levels compared to healthy controls [43]. It is worth mentioning that this study focused solely on the 677C>T polymorphism and did not research other polymorphisms of the *MTHFR* gene (such as the 1298A>C polymorphism), since there was no proven relation of the *MTHFR* gene polymorphisms to thyroid cancer, hyperhomocysteinemia or hypothyroidism, except for 677C>T, as previously stated. The present study revealed that the 677C>T polymorphism is distributed in a distinct pattern among children and adolescents with HT compared to healthy controls, adding new evidence to the underlying genetic milieu of the disease.

Many researchers in the previous decades demonstrated the increased susceptibility to thyroid cancer in patients with HT [17]. The findings of this study suggest that the 677C>T polymorphism of the *MTHFR* gene is a common factor of the aforementioned diseases. Due to the increased risk of developing thyroid cancer in patients with HT [46], which occurs with increasing incidence in the pediatric population [24], the need for prevention and early diagnosis is imperative.

Moreover, this study’s findings suggest that anti-TG are more frequently abnormal in patients with HT that are also carriers of the 677C>T polymorphism. This is also the case in patients with thyroid cancer, as suggested by Xiao et al. [47], who performed a meta-analysis of 12 independent studies and showed that anti-TG are an independent risk factor, with almost a twofold increase in risk for thyroid cancer.

The present original case–control study investigated for the first time the correlation of the 677C>T polymorphism of the *MTHFR* gene in children and adolescents and HT compared with healthy children and adolescents. Furthermore, secondary objectives such as gender, age, thyroid hormones and thyroid autoantibodies were examined for their possible independent effect on the frequency of the 677C>T polymorphism in children and adolescents with HT. All molecular methods applied in the present original study (gDNA isolation, PCR multiplication, RFLP method) strictly followed the recommended international guidelines and procedures. Prior to analysis, the power calculation performance predefined the adequacy of the investigated population.

The control group consisted of children and adolescents similar to those in the patient group. However, this does not exclude the possibility that some children and adolescents in the control group may develop HT in the future. This limits the results of the present study to the pediatric and adolescent population. The present study based the diagnosis of HT and the subsequent results on the autoantibodies of the thyroid gland (anti-TPO, anti-TG) and not on ultrasonographic findings. Although the complete description and diagnosis of Hashimoto’s thyroiditis includes ultrasonographic findings, these have not been incorporated in the present study because, on the one hand, there is great heterogeneity and subjectivity from different operators and, on the other hand, the ultrasound was performed at different time points of the disease. All of this could lead to incorrect assessments and unclear conclusions.

The study’s primary objective showed statistically significant results as the populations sample was based on this particular objective. However, some of the secondary objectives related to age and thyroid autoantibodies showed an increasing trend in children and adolescents with TH who had the T allele sequences of the polymorphism under investigation, but without a statistically significant difference. This could probably be explained by the relatively small sample size of the subgroups in the patient group and would probably not have occurred in a larger sample of the patient group.

More studies are needed to verify the findings regarding the association between the 677C>T polymorphism of the *MTHFR* gene and HT and to further explore the potential role of the 677C>T polymorphism in the occurrence of thyroid autoimmunity. Further verification of the findings of this study concerning the association between HT and the 677C>T polymorphism of the *MTHFR* gene raises a reasonable question for exploiting this particular polymorphism in children and adolescents with HT as a biomarker, resulting in more careful and regular screening and, ultimately, early detection of thyroid cancer.

## 5. Conclusions

Children and adolescents with HT have a 2.5-fold increased frequency of T allele sequences (CT and TT variations) of the 677C>T polymorphism (rs1801133) of the *MTHFR* gene compared to the healthy population. Additionally, T alleles within the 677C>T polymorphism were also 2.5 times more frequent in children and adolescents with HT compared to the healthy population. Finally, anti-TG autoantibodies were two times more frequent in children and adolescents with HT and with T allele sequences (CT and TT variation) compared to the wild-type (CC) sequence in the same population. More studies are needed to verify the findings of this study.

## Figures and Tables

**Table 1:** Baseline characteristics of the participants.

Categories	Control Group	Patient Group	*p*-Value
Total Participants	130	130	
Gender			
Male	41 (32%)	36 (27.7%)	-
Female	89 (68%)	94 (72.3%)	0.497
Age groups			
4–10 years	60 (46.1%)	67 (51.7%)	-
11–18 years	70 (53.9%)	63 (48.3%)	0.385
Age (mean value in years)	10.50	9.98	0.308
Laboratory findings			
TSH (μΙU/mL, n.r. 0.27–4.20)	2.59	2.87	0.218
fT4 (ng/dL, n.r. 0.98–1.63)	1.32	1.37	0.300
Anti-TPO (IU/mL, n.r. <34)	11.67	119.14	<0.001
Anti-TG (IU/mL, n.r. <115)	15.31	244.37	<0.001

Abbreviations: n.r.: normal range, TSH: thyroid stimulating hormone, fT4: free thyroxine, Anti-TPO: thyroid peroxide autoantibodies, Anti-TG: thyroglobulin autoantibodies.

**Table 2:** The association of the 677C>T polymorphism of the *MTHFR* gene in children and adolescents with HT vs. healthy population.

Genotype of 677 C>T	Patients (*n* = 130)	Controls (*n* = 130)	OR	CI (95%)	*p*-Value
CC	50 (38.4%)	80 (61.6%)	0.39	0.23–0.64	<0.001
CT	65 (50%)	41 (31.5%)	2.17	1.31–3.59	0.002
TT	15 (11.6%)	9 (6.9%)	1.75	0.73–4.16	0.20
CT and TT vs. CC	80 (61.6%)	50 (38.4%)	2.56	1.55–4.22	<0.001
C allele *	165	201	0.49	0.21–1.15	0.10
T allele *	95	59	2.57	1.59–4.16	<0.001

Abbreviations: OR: odds ratio, CI: confidence interval. * Total alleles were calculated to measure the effect of each allele independently. The total number of alleles was calculated by counting 2 C alleles in the CC variant (wild type), 1 C and 1 T allele in the heterozygous T allele sequence (CT variant) and 2 T alleles in the homozygous T allele sequence.

**Table 3:** The association of the 677C>T polymorphism of the *MTHFR* gene and the titer of thyroid autoantibodies in children and adolescents with HT.

Autoantibodies of the Thyroid	Genotype of the 677C>T Polymorphism	Number of Patients (*n*)	Mean Value	SD	SE	One-Way ANOVA (*p*-Value) *
Anti-TPO	CC	50	150.79	189.43	26.79	-
	CT	65	135.37	134.96	16.74	-
	TT	15	207.68	265.21	68.47	-
	Total	130	175.48	175.48	15.39	0.357
Anti-TG	CC	50	759.87	759.87	107.46	-
	CT	65	811.02	811.02	100.59	-
	TT	15	354.84	354.84	91.62	-
	Total	130	750.48	750.48	65.82	0.643

Abbreviations: Anti-TPO: thyroid peroxide autoantibodies, Anti-TG: thyroglobulin autoantibodies. * Comparison of anti-TPO and anti-TG variables among the subgroups of 677C>T polymorphism using one-way ANOVA statistical test.

**Table 4:** The association of the 677C>T polymorphism of the *MTHFR* gene and thyroid autoantibodies as dichotomous values (positive vs. negative titers) in children and adolescents with HT.

Autoantibodies of the Thyroid Gland	Genotype of 677C>T Polymorphism	Negative Autoantibody Titers	Positive Autoantibody Titers	Odds Ratio	CI 95%	*p*-Value
Anti-TPO	CC	4	46	0.37	0.11–1.20	0.100
	CT	13	52	2.45	0.87–6.93	0.089
	TT	19	13	0.88	0.18–4.28	0.881
	CT and TT	15	65	2.65	0.82–8.51	0.100
Anti-TG	CC	30	20	2.13	1.04–4.38	0.038
	CT	28	37	0.64	0.32–1.29	0.220
	TT	5	10	0.49	0.15–1.52	0.219
	CT and TT	33	47	0.46	0.22–0.96	0.038

Abbreviations: CI: Confidence interval, Anti-TPO: thyroid peroxide autoantibodies, Anti-TG: thyroglobulin autoantibodies.

## Data Availability

The data that support the findings of this study are not publicly available due to containing sensitive information that could compromise the privacy of research participants but are available from the corresponding author [Assimina Galli-Tsinopoulou] [E-mail address: agalli@auth.gr and gallitsin@gmail.com] upon reasonable request.

## Supplementary Materials

The following supporting information can be downloaded at https://www.mdpi.com/article/10.3390/diagnostics15111310/s1, Tables S1–S5. Additional Document. Table S1: Linear Regression Coefficients for Hashimoto’s Thyroiditis. Table S2: Logistic Regression Model to assess the effect of covariates on HT in children and adolescents. Table S3: The association of the 677C>T polymorphism of the MTHFR gene and age (mean value in years) in children and adolescents with HT. Table S4: The association of the 677C>T polymorphism of the MTHFR gene and age (age groups) in children and adolescents with HT. Table S5: The association of the 677C>T polymorphism of the MTHFR gene and age (age groups) in children and adolescents with HT.

## Abbreviations

The following abbreviations are used in this manuscript:

HT	Hashimoto’s thyroiditis
MTHFR	Methylenetetrahydrofolate reductase
Anti-TPO	Thyroid peroxide autoantibodies
Anti-TG	Thyroglobulin autoantibodies
DNA	Deoxyribonucleic acid
STROBE	Strengthening the Reporting of Observational Studies in Epidemiology
ECLIA	Electrochemiluminescent immunoassays
gDNA	Genomic Deoxyribonucleic acid
RFLP	Restriction Fragment Length Polymorphism
PCR	Polymerase Chain Reaction
GAS	Genetic Association Study
Nr	Normal Range
TSH	Thyroid stimulating hormone
fT4	fT4: Free thyroxine
OR	Odds ratio
CI	Confidence Interval
