# Supplementary material for: Study of the MTHFR 677C>T Polymorphism in Children and Adolescents with Hashimoto’s Thyroiditis: An Original Case–Control Study
Source: Diagnostics (Basel). 2025 May 23;15(11):1310. doi: 10.3390/diagnostics15111310 (PMC12154079; doi:10.3390/diagnostics15111310)
Supplement: Supplementary file 1 [file diagnostics-15-01310-s001.zip › diagnostics-3596562-supplementary.pdf]

Supplementary Material

Table S1: Linear Regression Coefficients for Hashimoto's Thyroiditis

Variable	Standardized Coefficients Beta	SE	p-value
677C>T Polymorphism (additive model)	0.149	0.042	0.007
Gender	0.037	0.058	0.497
Thyroid Status	0.093	0.085	0.117
Age (in years)	-0.133	0.014	0.196
TSH (μIU/mL)	-0.054	0.016	0.343
fT4 (ng/dL)	-0.056	0.085	0.325
Anti-TPO (IU/ml)	0.407	0.000	<0.001
Anti-TG (IU/ml)	0.121	0.000	0.053

Abbreviations: TSH: thyroid stimulating hormone, fT4: free thyroxine, Anti-TPO: thyroid peroxide autoantibodies, Anti-TG: thyroglobulin autoantibodies, SE: Standardized Error

Table S2: Logistic Regression Model to assess the effect of covariates on HT in children and adolescents

Variable	S.E.	CI (95%)	p-value
677C>T Polymorphism	0.205	1.355–3.030	<0.001
Age	0.038	0.891–1.032	0.266
Gender	0.280	0.539–1.617	0.806
TSH (μIU/mL)	0.135	0.925–1.570	0.166
fT4 (ng/dL)	0.139	0.851–1.466	0.426

Abbreviations: TSH: thyroid stimulating hormone, fT4: free thyroxine, CI: Confidence interval, S.E: Standard Error

Table S3: The association of the 677C>T polymorphism of the *MTHFR* gene and age (mean value in years) in children and adolescents with HT

Genotype of the 677C>T polymorphism	Number of children and adolescents	Age (mean value in years)	SD	CI (95%)	One-way ANOVA
CC	50	9.18	3.49	8.19 – 10.17	-
CT	65	9.09	3.31	8.27 – 9.91	-
TT	15	7.80	2.62	6.35 – 9.25	-
Total	130	8.98	3.31	8.40 – 9.55	0.343

Abbreviations: HT: Hashimoto's Thyroiditis, OR: odds ratio, CI: Confidence interval

Table S4: The association of the 677C>T polymorphism of the *MTHFR* gene and age (age groups) in children and adolescents with HT

Genotype of the 677C>T polymorphism	Age group of 4-10 years old (n= 67)	Age group of 11-18 years old (n= 63)	OR	CI (95%)	P value
CC	29	21	1.52	0.74 - 3.11	0.240
CT	27	38	0.44	0.22 – 0.89	0.023
TT	11	4	2.89	0.87 – 9.36	0.082
CT & TT vs CC	38	42	0.65	0.32 – 1.33	0.240

Abbreviations: HT: Hashimoto's Thyroiditis, OR: odds ratio, CI: Confidence interval

Table S5: The association of the 677C>T polymorphism of the *MTHFR* gene and age (age groups) in children and adolescents with HT

Genotype of the 677C>T polymorphism	Patients' Group			Controls' Group			OR		CI		P value	
	Male	Female	Total	Male	Female	Total	Male	Female	Male	Female	Male	Female
CC	16	34	50	27	53	80	0.43	0.378	0.17 – 1.10	0.20 – 0.68	0.797	0.001
CT	16	49	65	12	29	41	2.03	1.29	0.79 – 5.24	0.71 – 2.35	0.141	0.396
TT	3	12	15	2	7	9	1.82	1.69	0.28 – 11.61	0.63 – 4.51	0.522	0.292
CT&TT vs CC	19	51	80	14	36	50	2.29	2.64	0.90 – 5.78	1.45 – 4.79	0.079	0.001

Abbreviations: HT: Hashimoto's Thyroiditis, OR: odds ratio, CI: Confidence interval
